# Novel *SIAH1* Frameshift Variant in a Chilean Patient With Buratti–Harel Syndrome

**DOI:** 10.1155/crig/8822406

**Published:** 2025-10-27

**Authors:** Nicole Nakousi C., Catalina Nakousi M., Gabriela Perez C.

**Affiliations:** ^1^Pediatrics Service, Carlos Van Buren Hospital, Valparaíso, Chile; ^2^Facultad de Medicina, Universidad Finis Terrae, Santiago, Chile; ^3^Child Neurology Department, Pediatrics Service, Carlos Van Buren Hospital, Valparaíso, Chile

**Keywords:** Buratti–Harel syndrome, BURHAS, de novo variant, exome sequencing, genetic diseases, next generation sequencing, *SIAH1* gene

## Abstract

Buratti–Harel syndrome (BURHAS) is a rare genetic condition caused by heterozygous pathogenic variants of the *SIAH1* gene, with only five unrelated cases included in a single report in 2019. BURHAS is characterized mainly by neurodevelopmental delay, infantile hypotonia, and dysmorphic features. We report the case of a 9-year-old Chilean female that matches this phenotype, with a heterozygous, *de novo* frameshift variant of the *SIAH1* gene.

## 1. Introduction

We report the case of a Chilean 9-year-old girl presenting with congruent phenotypic characteristics: mild neurodevelopmental delay, dysmorphic features, infantile hypotonia, and gastroesophageal reflux with a novel heterozygous, *de novo* variant in the seven in absentia homolog 1 (*SIAH1*) gene.

## 2. Case Report

We present a 9-year-old female, second-born child to parents of Latin American descent. Parents were healthy and nonconsanguineous, with no relevant family medical history. The patient was born full term via caesarean section after a 38-week gestation, following an uneventful pregnancy and delivery. Birth weight was 3390 g, and birth length was 49 cms, both within normal range for locally-adjusted values [1].

At 10 months old, during an extended hospital stay for a respiratory viral infection, the patient was evaluated for dysmorphic features and failure to thrive and referred to neurology for further assessment, where the parents recount the child was described as “extremely calm, did not reach for objects or reposition readily.” Parents also reported she presented mild gastroesophageal reflux during early infancy, which did not receive treatment and resolved spontaneously.

Recovered data from early physical evaluations reveal a progressive height stunting in height-to-age curves between 0 and 36 months of age [2], even falling to a z-score of −2 SD at 24 months of age, equivalent to the 0.8th percentile. There was no additional anthropometric data available.

Follow-up evaluation by neurology at 9 years of age revealed moderately impaired intellectual development with learning difficulties and attention deficit hyperactivity disorder. The patient presented a delay in achieving motor and speech developmental milestones, sitting up with no support at around 6 months old, and walking on her own not before 2 years of age, for which she required extensive neurorehabilitation. The child expressed her first identifiable words at 20 months and achieved urinary continence at 42 months.

During this evaluation, she presented axial hypotonia, joint hypermobility, and indifferent left plantar reflex with normal osteotendinous reflexes. She was able to keep up with conversation, only presenting mild difficulties with grammatical structure and pronunciation. Parents reported the patient was able to eat and get dressed on her own but needed help with personal hygiene, and that she required curricular adaptation at school because of her learning difficulties.

The child was referred to genetics at 9 years and 1 month of age, where her weight was 38.500 kg (z-score of +2 SD for BMI adjusted by age) and height was 123 cm (z-score of −1 SD for height adjusted by age), both values were determined by growth curves based on WHO Child Growth Standards [2]. These values reflect mild height stunting and obesity.

**Figure 1:**
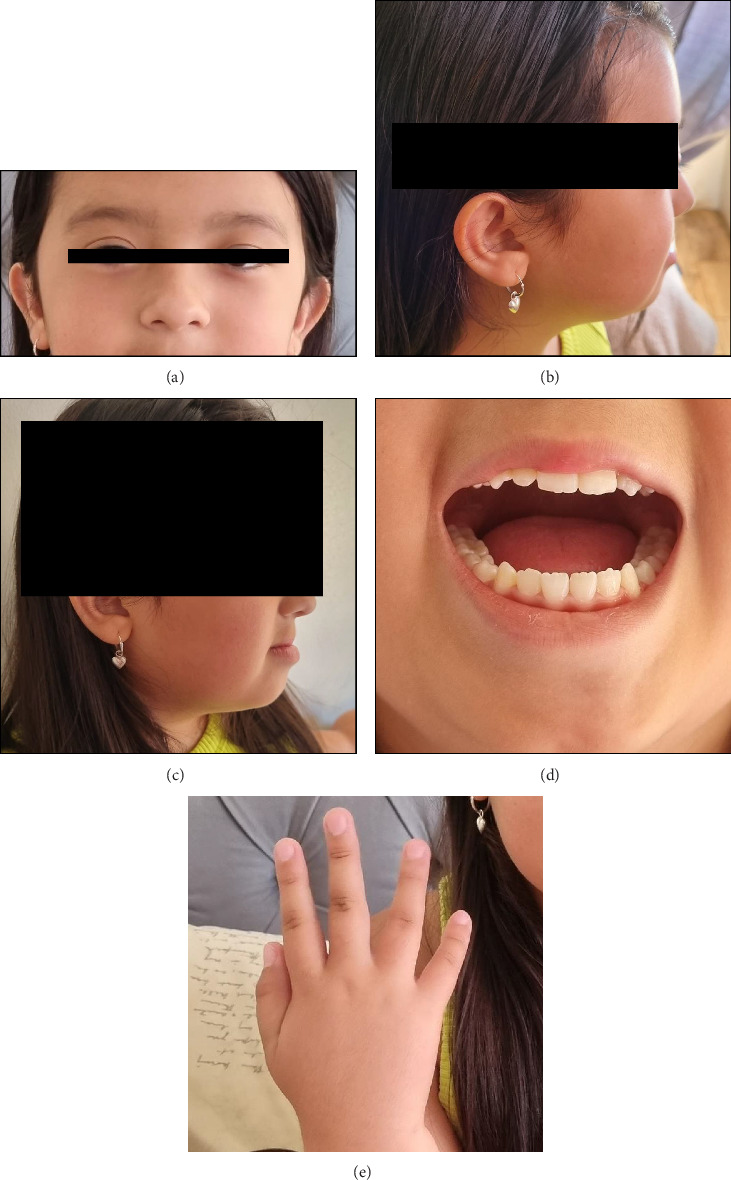
Physical examination findings: (a) Hypertelorism, short, upslanted palpebral fissures and low-set ears. (b) Depressed middle third of the face and flattened facial profile. (c) Prognathism. (d) Serrated teeth. (e) Mild brachydactyly and fifth finger clinodactyly.

Clinical features described at physical examination in this occasion included the following: flat facial appearance, hypertelorism, small eyes, upslanted palpebral fissures, low-set ears, serrated teeth, prognathism, thin neck, clinodactyly of fifth fingers, and feet with mild fallen arches (Figure 1). Cardiopulmonary exploration and abdominal exploration were within normal limits. An echocardiogram showed a persistent ductus arteriosus at 9 years old. A brain CT had been performed, with normal results according to parents, though no formal report was obtained. She has a blood karyotype 46,XX.

Further testing included thyroid function evaluation, which revealed thyroid-stimulating hormone (TSH) levels of 1.69 uUI/mL and free thyroxine (FT4) levels of 1.32 uUI/mL. However, autoantibody levels were significantly elevated, with anti-thyroid peroxidase antibody (anti-TPO) at 1177.01 UI/mL and anti-thyroglobulin antibody (TgAbs) at 2354.42 UI/mL. She is under supervision by a pediatric endocrinologist for this motive and has not required pharmacological treatment so far.

Exome sequencing (ES) was performed at an external laboratory and reported a heterozygous frameshift variant of uncertain significance in the *SIAH1* gene (NM_001006610.1:c.116dup, p.[Leu40Ilefs∗26]). This variant was not found in healthy individuals in gnomAD genomes (gnomAD genomes, despite a good coverage = 31.3) [3] and is expected to truncate Siah E3 ubiquitin protein ligase 1 at Amino acid 66. Sanger sequencing was locally performed on both parents, and the *de novo* status of the variant was confirmed.

## 3. Discussion

Buratti–Harel syndrome (BURHAS) was first described in 2019, when a single publication reported five unrelated individuals, aged between 18 months and 15 years, evaluated for developmental delay, infantile hypotonia, and dysmorphic features. Trio ES followed by Sanger validation yielded monoallelic *de novo* variants in the *SIAH1* gene in each individual [4].

SIAHs are a family of highly conserved E3 ubiquitin ligases that play important roles in different pathways, for example, the regulation of Wnt/β-catenin signaling and Axin ubiquitination [5]. Wnt signaling must be tightly regulated in order to mediate embryonic patterning, cell polarity, and cell specification. During nervous system development, Wnt proteins mediate neuronal differentiation, axonal outgrowth and guidance, dendrite development, synaptic function, and neuronal plasticity [6].

*SIAH1*'s influence in neuronal function extends beyond the β-catenin pathway and acts via synaptophysin, α-synuclein, and synphilin-1 to modify presynaptic proteostasis and neuronal development [7]. It is also described as a regulator of metabotropic glutamate receptor signaling and ubiquitin-mediated degradation of Akt3 during neural development, disrupting the organization of neural networks [8]. Furthermore, animal models have shown that *SIAH1* regulates timely radial migration, neural cell fate, and neuronal polarity [9, 10].

Loss of function of *SIAH1* has been suggested as the mechanism underlying BURHAS [4, 11]. While we recognize functional studies of our patient's variant are still needed to establish causality in our patient, it is located on a conserved nucleotide in Exon 2, creating a stop codon of 26 amino acids downstream and truncating its protein's RING-type zinc finger domain. This domain has been shown in vitro to be important for Wnt stimulatory activity in the first reports of BURHAS [4]. Those cases reported missense variants in the RING domain (*n* = 2), zinc finger motif (*n* = 1), and further downstream (p.Thr168Ala and p.Gly174Arg), but no truncating variants [4]. A recent report of eight individuals with a BURHAS phenotype described five truncating or nonsense variants upstream or within this RING-type domain. The authors did not perform RNA sequencing or other functional testing, but expected them to lead to a loss of key functional sequences, predicting a partial or complete loss of *SIAH* E3 ubiquitin protein ligase 1 activity and classified them as pathogenic or likely pathogenic. Half of these variants truncated the protein between Amino acids 33 and 35 (RING domain), and the farthest one was localized at Position 118 (zinc finger domain). Unclustered, missense variants were reported in two patients, one in the zinc finger domain (p.His152Arg) and the other further downstream (p.Asp177His) [12].

Until the moment of this report, 70 germline variants in *SIAH1* can be found in ClinVar. They include all twelve patient reports mentioned [12]. Among those that are pathogenic (nineteen in total), nine are part of copy number gains, five are number losses, and four are missense. The aforementioned CNVs include other genes and are not phenotypically associated with BURHAS. The twelve likely pathogenic variants cause mostly missense and frameshift changes (*n* = 5 each), followed by two nonsense SNVs. All are located in Exon 2 of *SIAH1*. While haploinsufficiency of *SIAH1* has been argued as a mechanism of action so far [4, 12], the fact that most pathogenic SNVs associated with BURHAS are missense may argue in favor of a dominant negative effect as a possible mechanism of action in this syndrome.

In spite of nonsense-mediated decay not being expected in our patient 's variant, given its localization in the second exon of *SIAH1*, it is expected to modify Amino acid 40 (Leu) for Ile and interrupt the Protein 26 positions downstream at Amino acid 66. This is within the region where likely pathogenic/pathogenic truncating variants for BURHAS are present in public repositories [4, 12, 13]. It has not been described previously in the general population nor in BURHAS patients, and similar to them, it occurred *de novo* [3]. While initially classified as a VUS, we believe these are arguments in favor of a likely pathogenic classification by fulfilling criteria PM1, PM2, and PS2 of the American College of Medical Genetics and Genomics [14].

The characteristics of our patient, which are shared with at least 8 out of 13 of all individuals described [4, 12], include the following: infantile hypotonia, laryngomalacia, GERD, speech and motor delay, intellectual disability, and dysmorphism. Hypertelorism, medially sparse eyebrows, and small, low-set ears are features our patient shares with four out of five of the individuals originally reported [4] (Table 1).

Upslanting palpebral folds and serrated tooth borders in our patient do not seem to contradict the original reports and might contribute to expanding its phenotype. Until now, dental anomalies have only been recently described and include oligodontia in two individuals, hypoplastic teeth in one individual and unusual upper central incisors with prominent central mamelons in one individual [12]. This last element was observed in our patient, giving the appearance of serrated tooth borders in upper and lower incisors but may not be specific to BURHAS, as they are frequently persistent in people with misaligned teeth or open bites that impede their normal contact. In this case, it may be related to her prognathism.

It is worth commenting on our patient's elevated anti-TPO and TgAbs levels in the context of euthyroid thyroiditis. To our knowledge, only one individual with BURHAS showed transient neonatal hypothyroidism. They were followed until the age of 12 years and 11 months [12]. Another presented Type 1 diabetes mellitus at 13 years old. However, autoimmune thyroid disease is also prevalent in the pediatric population [15, 16], and our patient's maternal grandmother suffers from systemic lupus erythematosus (SLE), which may increase her predisposition to autoimmune disorders. We therefore believe it may not necessarily be related to BURHAS syndrome, but recognize that more descriptions are needed.

Limitations of our report include the following: a variant classification of uncertain significance due to the absence of functional studies and also phenotypic variability of BURHAS that may explain why our patient presents additional phenotypic characteristics that are not clearly understood for now.

## 4. Conclusion

This is the first complete description of a phenotype and genotype of a patient with a novel heterozygous frameshift variant in *SIAH1*, and a first report of a Chilean individual with developmental delay, infantile hypotonia, and facial dysmorphism. Our clinical findings are in correspondence with what has already been described for BURHAS. While we believe further functional studies of this variant would be useful to describe its consequences on Wnt signaling and causality, we hope this report is useful in expanding the phenotypic characterization in the Latin American population.

We propose ES as a first-line study in cases of nonspecific dysmorphic features, neurodevelopmental delay, and infantile hypotonia in order to expedite their diagnostic process. Reclassification of variants of uncertain significance may be possible by profound clinical characterization, genetic testing of parents, and revision of public databases and functional studies. These are highly needed for *SIAH1*, since the mechanism of action through which heterozygous variants cause BURHAS remains unclear, with some evidence of haploinsufficiency but without the possibility of ruling out dominant negative effects.

## Figures and Tables

**Table 1:** Characteristics of patients described in previous reports (Buratti et al., 2021 and Douiev et al., 2025) as compared to our patient.

	This report (1 individual)	Buratti et al., 2021 (*n* = 5)	Douiev et al., 2025 (*n* = 8)
Infantile hypotonia	+	5	4
Laryngomalacia	−	4	5
GERD	+	4	4
Recurrent pneumonia	−	2	3
Serous otitis media	−	2	N/A
Motor delay	+	4	8
Speech delay	+	5	7
Intellectual disability	+ (Global, mild to moderate)	3	6
Cardiac malformations	+ (Persistent ductus arteriosus)	1	4
Ophthalmological abnormalities	−	3	N/A
Endocrinologic abnormalities	+ (Euthyroid thyroiditis)	N/A	3
Dysmorphic features	+	5	7
Epicanthal folds	−	4	N/A
Hypertelorism	+	4	N/A
Deviated palpebral fissures	Upslanting	Downslanting (3)	N/A
Medially sparse eyebrows	+	5	N/A
Cleft palate	−	1	5
Mandible	Prognathism	Micrognathia (2) prognathism (1)	N/A
Small, low-set, posteriorly rotated ears	+	5	N/A
Broad thumbs/hallux	N/A	2	N/A
Fifth finger clinodactyly	+	4	N/A
*SIAH1* variant(s)	(NM_001006610.1) c.116dup/ p.(Leu40Ilefs∗26)	(NM_003031.4) c.502A > G/p.(Thr168Ala) c.383G > T/p.(Cys128Phe) c.121T > G/p.(Cys41Gly) c.520G > C/p.(Gly174Arg) c.149C > T/p.(Pro50Leu)	(NM_003031.4) c.15_16insA/p.(Ala6Serfs29∗) c.19dup/p.(Thr7Asnfs∗28) c.71_75del/p.(Ala24Aspfs∗9) c.104T > A/p.(p. Leu35Ter) c.165T > A/p.(Cys55Ter) c.337_338del/p.(Glu113Lysfs∗5) c.455A > G/p.(His152Arg) c.529G > C/p.(Asp177His)

*Note:* N/A, data not available.

Abbreviations: GERD, gastroesophageal reflux disease.

## Data Availability

The data that support the findings of this study are available from the corresponding author upon reasonable request.
